# Insight into the Molecule Impact of Critical-Sized UHMWPE-ALN Wear Particles on Cells by the Alginate-Encapsulated Cell Reactor

**DOI:** 10.3390/ijms24043510

**Published:** 2023-02-09

**Authors:** Yumei Liu, Feng Shi, Shuxin Qu

**Affiliations:** 1 Collaboration and Innovation Center of Tissue Repair Material Engineering Technology, College of Life Science, China West Normal University, Nanchong 637009, China; 2 College of Environmental Science and Engineering, China West Normal University, Nanchong 637009, China; 3 Key Laboratory of Advanced Technologies of Materials (Ministry of Education), School of Materials Science and Engineering, Southwest Jiaotong University, Chengdu 610031, China

**Keywords:** critical size, UHMWPE-ALN wear particles, alginate-encapsulated, cell reactor, molecular mechanism

## Abstract

Wear particles of ultra-high molecular weight polyethylene (UHMWPE) are inevitable during service as joint prosthesis, and particles ≤ 10 μm with critical size could cause serious osteolysis and aseptic loosening of joint prosthesis. The aim of this study is to adopt the alginate-encapsulated cell reactor to investigate the molecular impact of critical-sized wear particles of UHMWPE loaded with alendronate sodium (UHMWPE-ALN) on cells. Results showed that compared with UHMWPE wear particles, UHMWPE-ALN wear particles inhibited the proliferation of macrophages significantly after being co-cultured for 1, 4, 7, and 14 d. Furthermore, the released ALN promoted early apoptosis, suppressed the secretion of TNF-α and IL-6 of macrophages, and down-regulated relative gene expressions of TNF-α, IL-6, and IL-1β and RANK. In addition, compared with UHMWPE wear particles, UHMWPE-ALN wear particles promoted the ALP activity of osteoblasts, down-regulated the gene expression of RANKL, and up-regulated gene expression of osteoprotegerin. There were mainly two approaches of the effects of critical-sized UHMWPE-ALN wear particles on cells, one of which was cytology and the other was cytokine signal pathway. The former mainly affected the proliferation and activity of macrophages and osteoblasts. The latter would inhibit osteoclasts via cytokine and RANKL/RANK signal pathway. Thus, UHMWPE-ALN had the potential application in clinics to treat osteolysis induced by wear particles.

## 1. Introduction

Ultra-high molecular weight polyethylene (UHMWPE) has been successfully used as a bearing material for joint replacement prostheses for more than half century [1]. Nevertheless, critical-sized UHMWPE wear particles, that are less than 10 μm in size, are currently recognized as one of the major factors that induce osteolysis and aseptic loosening of joint replacement prostheses in clinics [2,3,4]. To date, a number of studies have proposed some useful strategies to improve the wear resistance of UHMWPE [5,6,7,8]. However, the wear particles of UHMWPE are still inevitable. Alendronate sodium (ALN), one kind of nitrogen-containing bisphosphonate, has been widely used to treat bone resorption and wear particle-induced osteolysis in clinic [9,10]. In our previous study, 0.5 wt.% ALN loaded in UHMWPE (UHMWPE-ALN) has been optimized and prepared to study the release of ALN and the impact of critical-sized UHMWPE-ALN wear particles on macrophages in vitro [7,11]. Critical-sized UHMWPE-ALN wear particles could release partial ALN in vitro and subsequently reduce the proliferation and induce the lactate dehydrogenase (LDH) leakage of macrophages after being co-cultured [12]. Moreover, the evidence confirms that macrophages can be chemotaxis and aggregated around the critical-sized UHMWPE-ALN wear particles, phagocytose them and secrete some cytokines, e.g., interleukin-6 (IL-6) and tumor necrosis factor-alpha (TNF-α). In addition, the secretion of those cytokines by macrophages are reduced significantly after co-cultured with critical-sized UHMWPE-ALN wear particles compared to those with critical-sized UHMWPE wear particles [12]. However, it is hard to co-culture cells and UHMWPE wear particles for a relatively long time due to the difficulty of replacing cell culture medium in an inverted culture technique and by other co-culture approaches [13,14,15,16].

In recent years, the use of different polymers to form cell-coated hydrogels for tissue repair has become a hot topic and has a good application prospect [17,18]. In our previous study, a novel alginate (Alg)-encapsulated bead was developed to co-culture UHMWPE-ALN wear particles and osteoblasts [19]. Alg-encapsulated beads can act as a cell reactor to co-culture cells and UHMWPE-ALN wear particles. Osteoblasts and critical-sized UHMWPE-ALN wear particles distributed evenly and contacted efficiently in Alg-encapsulated beads. Particularly, this Alg-encapsulated cell reactor is feasible for evaluating the biological response of cells to critical-sized UHMWPE-ALN wear particles for a relatively long time. However, only the cell viability and alkaline phosphatase (ALP) of osteoblasts are preliminarily evaluated after being co-cultured with critical-sized UHMWPE-ALN wear particles in the Alg-encapsulated bead for a relatively long time.

It is strong evidence that there are several molecule pathways for the impact of UHMWPE wear particles on wear particle-induced osteolysis. As an important part of the immune system, macrophages can be stimulated by biological materials, thus changing their phenotypes and further affecting the physiological functions of tissues [20]. Therein, macrophages play the primary role by secreting a series of cytokines after phagocytosis of critical-sized UHMWPE wear particles and further result in the maturation of osteoclasts through the cytokine pathway [4,13]. Furthermore, UHMWPE wear particles can inhibit the osteogenic activity of osteoblasts and enhance the maturation of osteoclasts through the receptor activator of nuclear factor kappa-B (RANK)/RANK ligand (RANKL) signal pathway. However, the detailed molecule pathways of critical-sized UHMWPE-ALN wear particles on macrophages and osteoblasts are still not clear to date. Moreover, there is still partial ALN that remains in critical-sized UHMWPE-ALN wear particles, which may be phagocytosed by macrophages or directly have influence on both macrophages and osteoblasts. Therefore, it is necessary to reveal the molecule impact mechanisms of critical-sized UHMWPE-ALN wear particles on both macrophages and osteoblasts for the further clinical application.

In this study, an Alg-encapsulated bead was adopted as the cell reactor to evaluate the response of macrophages and osteoblasts to critical-sized UHMWPE-ALN wear particles. Moreover, the molecule impact mechanisms of critical-sized UHMWPE-ALN wear particles on macrophages and osteoblasts were investigated in vitro.

## 2. Results

### 2.1. Biological Evaluation of Critical-Sized UHMWPE-ALN Wear Particles on Macrophages Encapsulated in Cell Reactors

#### 2.1.1. Alamar Blue Assay

**Figure 1:**
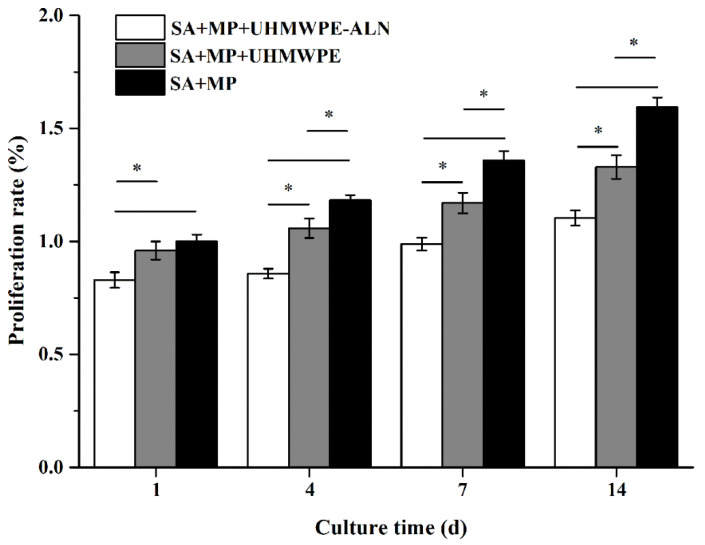
The relative proliferation rates of macrophages after being co-cultured with critical-sized UHMWPE-ALN wear particles in cell reactors for 1, 4, 7, and 14 d (*n* = 3, * *p* < 0.05).

Figure 1 shows the relative proliferation rates of macrophages after being co-cultured with critical-sized UHMWPE-ALN wear particles in cell reactors for 1, 4, 7, and 14 d. The relative proliferation rates of macrophages in cell reactors increased gradually with the co-culturing time. Notably, the relative proliferation rates of macrophages in Alg + MPs + UHMWPE-ALN group were significantly lower than those in Alg + MPs + UHMWPE group after being co-cultured for 1, 4, 7, and 14 d.

#### 2.1.2. Cell Apoptosis Assay

**Figure 2:**
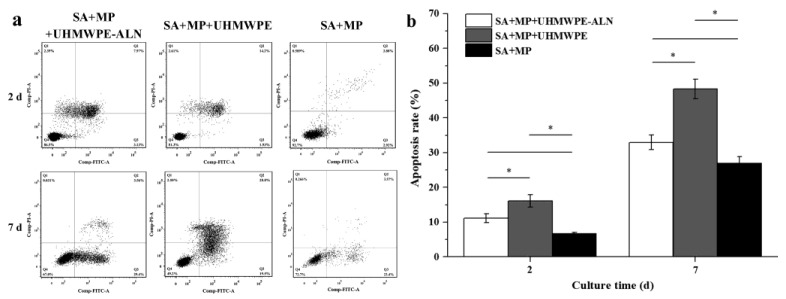
Effects of critical-sized UHMWPE-ALN wear particles on the apoptosis of macrophages in cell reactors after incubated for 2 and 7 d. (**a**): The representative images of flow cytometry. Q1 (Annexin V-FITC-/PI+): percentage of necrosis cells; Q2 (Annexin V-FITC+/PI+): percentage of late apoptosis cells; Q3 (Annexin V-FITC+/PI-): percentage of early apoptosis cells; and Q4 (Annexin V-FITC-/PI-): percentage of viable cells. (**b**): Quantitative apoptosis rates of macrophages by flow cytometry (*n* = 3, * *p* < 0.05).

Figure 2 shows the apoptosis of macrophages in cell reactors after being co-cultured with critical-sized UHMWPE-ALN wear particles for 2 and 7 d by flow cytometry. In Figure 2a, there are four quadrants, upper left (Q1), upper right (Q2), lower right (Q3), and lower left (Q4) in each small graph. Figure 2b shows the quantitative apoptosis rates (Q2 + Q3) of macrophages in each group of cell reactors according to Figure 2a. The apoptosis rates of macrophages increased with time in all groups (Figure 2b). Furthermore, the apoptosis rates of macrophages co-cultured with wear particles were significantly higher than those of the cell reactor alone (*p* < 0.05), indicating that the addition of wear particles induced the apoptosis of macrophages. In addition, the early apoptosis rate of macrophages in the Alg + MPs + UHMWPE-ALN group was significantly higher than that in the Alg + MPs + UHMWPE group, which might be due to the release of ALN from critical-sized UHMWPE-ALN wear particles.

#### 2.1.3. Live-Dead Assay

**Figure 3:**
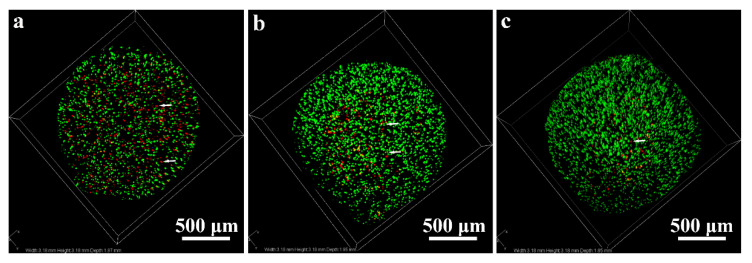
Confocal laser scanning microscope images of macrophages after being co-cultured with critical-sized wear particles in cell reactors for 2 d. (**a**) Alg + MPs + UHMWPE-ALN, (**b**) Alg + MPs + UHMWPE, and (**c**) Alg + MPs. White arrows indicate the dead macrophages.

Figure 3 shows the live-dead fluorescence-stained macrophages in cell reactors after being incubated for 2 d. The green fluorescence shows the live macrophages labeled by calcein-AM, and red fluorescence shows dead macrophages labeled by PI. A large number of live macrophages were observed in cell reactors. In addition, there were more dead macrophages labeled as red (indicated by white arrows) that appeared in the Alg + MPs + UHMWPE-ALN and Alg + MPs + UHMWPE groups after being co-cultured for 2 d compared with those in the Alg + MPs group (Figure 3c). Macrophages were distributed homogeneously in all cell reactors as shown in Figure 3.

#### 2.1.4. Cytokine Assay

**Figure 4:**
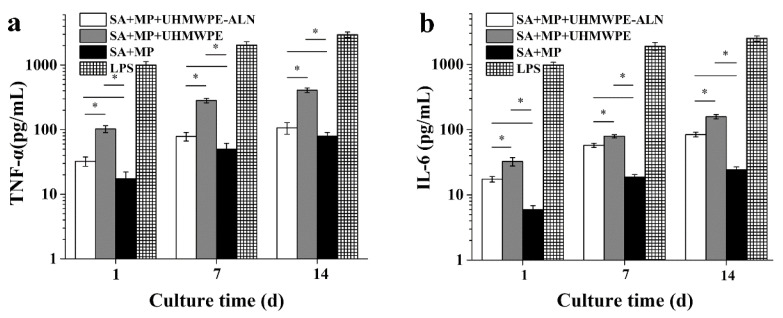
The secreted levels of TNF-α (**a**) and IL-6 (**b**) from macrophages after being co-cultured with critical-sized UHMWPE-ALN wear particles in cell reactors for 1, 7, and 14 d (*n* = 3, * *p* < 0.05). The level of IL-6 (**a**) or TNF-α (**b**) of each group at the same culture time was significantly lower than that of the LPS group (not marked).

Figure 4 shows the levels of cytokines secreted from macrophages after being co-cultured with critical-sized UHMWPE-ALN wear particles in cell reactors for 1, 7, and 14 d. The secreted levels of TNF-α (Figure 4a) and IL-6 (Figure 4b) of macrophages increased with time in different groups. Particularly, the secreted levels of TNF-α and IL-6 cytokines of macrophages in the Alg + MPs + UHMWPE-ALN and Alg + MPs + UHMWPE groups were significantly higher than those in the Alg + MPs group, which demonstrated that the critical-sized wear particles would stimulate the secretion of cytokines from macrophages, which was analogous to the findings of Li et al. [21]. However, the secreted levels of TNF-α and IL-6 cytokines from macrophages in the Alg + MPs + UHMWPE-ALN group were significantly lower than those in the Alg + MPs + UHMWPE group. In the positive lipopolysaccharide (LPS) group, the secreted levels of TNF-α and IL-6 of macrophages were significantly higher than those of other groups. In addition, Figure 4 shows that the secreted level of TNF-α from macrophages in each group were almost higher than those of IL-6 after being cultured in all groups.

#### 2.1.5. RT-PCR Analysis

**Figure 5:**
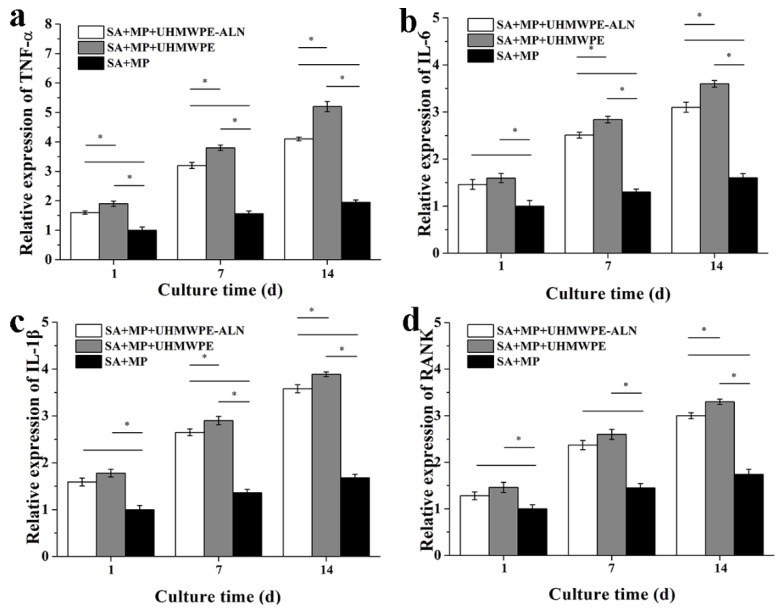
Relative gene expressions of TNF-α (**a**), IL-6 (**b**), IL-1β (**c**), and RANK (**d**) of macrophages after being co-cultured with critical-sized UHMWPE-ALN wear particles in cell reactors for 1, 7, and 14 d by RT-PCR analysis (*n* = 3, * *p* < 0.05).

Figure 5 shows the relative gene expressions of macrophage-related genes from macrophages after being co-cultured with critical-sized UHMWPE-ALN wear particles in cell reactors for 1, 7, and 14 d. The relative gene expression of macrophage-related genes, TNF-α, IL-6, IL-1β, and RANK, were all up-regulated with time for each group, with various levels. The fastest up-regulated gene expression of TNF-α was observed with time compared with other genes. The relative gene expressions of TNF-α, IL-6, IL-1β, and RANK were significantly higher in the wear particles group than those in the Alg + MPs group at the same time. Notably, the expressions of macrophage-related genes were lower after being co-cultured with UHMWPE-ALN wear particles compared with those with UHMWPE wear particles.

### 2.2. Biological Evaluation of Critical-Sized UHMWPE-ALN Wear Particles with Osteoblasts Encapsulated in Cell Reactors

#### 2.2.1. ALP Assay

**Figure 6:**
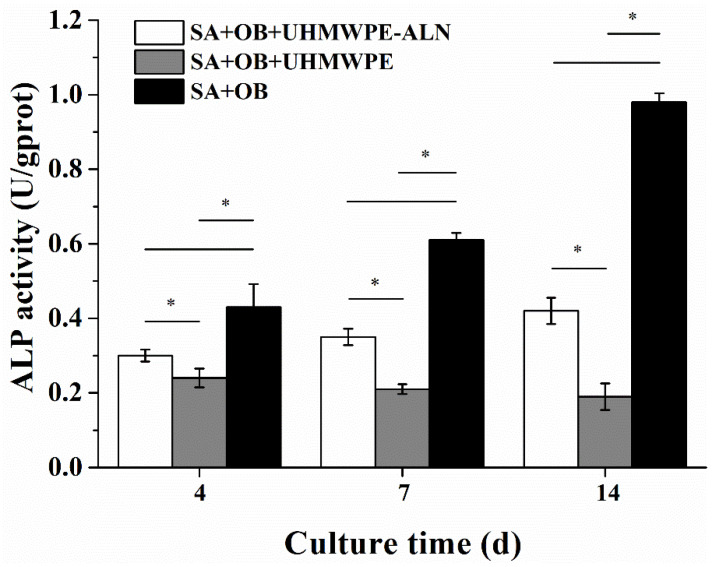
The ALP activities of osteoblasts after being co-cultured with critical-sized UHMWPE-ALN wear particles in cell reactors for 4, 7, and 14 d (*n* = 3, * *p* < 0.05).

Figure 6 shows the ALP activity of osteoblasts after being co-cultured with critical-sized UHMWPE-ALN wear particles in cell reactors for 4, 7, and 14 d. The ALP activity of each group was normalized to the corresponding total protein content in this study, which could avoid the difference due to the different number of cells in each group. The effect of wear particles on the differentiation of osteoblasts was reflected after normalization. The ALP activities of osteoblasts co-cultured with wear particles were significantly lower than those of the Alg + OBs group (*p* < 0.05). The ALP activities of osteoblasts co-cultured with UHMWPE-ALN wear particles increased gradually with time, while the ALP activities of osteoblasts co-cultured with UHMWPE wear particles showed a decreasing trend with time. The ALP activities in the Alg + OBs + UHMWPE-ALN group were significantly higher than those in the Alg + OBs + UHMWPE group at the same time.

#### 2.2.2. RT-PCR Assay

**Figure 7:**
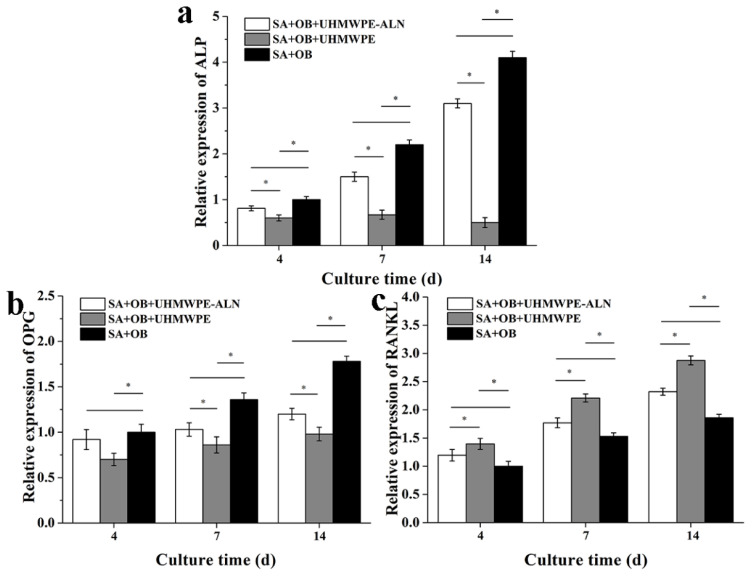
Relative gene expressions of ALP (**a**), RANKL (**b**), and OPG (**c**) in osteoblasts after being co-cultured with critical-sized UHMWPE-ALN wear particles for 4, 7, and 14 d by RT-PCR analysis (*n* = 3, * *p* < 0.05).

Figure 7 shows the relative gene expression of osteoblast-related genes, ALP, OPG, and RANKL, after being co-cultured in cell reactors for 4, 7, and 14 d. The up-regulated gene expression of ALP of osteoblasts appeared in the Alg + OBs + UHMWPE-ALN and Alg + OBs groups with time, while the down-regulated gene expression of ALP appeared in the Alg + OBs + UHMWPE group with time. In addition, the up-regulated gene expression of OPG and RANKL of osteoblasts appeared in all groups with time. Furthermore, the gene expression of ALP and OPG of osteoblasts were the highest in the Alg + OBs group and followed by the Alg + OBs + UHMWPE-ALN group. However, the gene expressions of RANKL of osteoblasts were the highest in the Alg + OBs + UHMWPE group and followed by the Alg + OBs + UHMWPE-ALN group.

## 3. Discussion

Alendronate sodium (ALN), one kind of nitrogen-containing bisphosphonate, has been widely used to treat bone resorption and wear particle-induced osteolysis in clinical studies [10,22]. In our previous study, ALN loaded in UHMWPE (UHMWPE-ALN) was developed for anti-osteolysis resulting from joint replacement prostheses. It is expected that the released ALN, with the inevitable UHMWPE-ALN wear particles, could decrease the biological response of cells to wear particles. It was investigated that in vitro release of ALN could subsequently reduce the biological response of macrophages after being co-cultured with critical-sized UHMWPE-ALN wear particles compared to those with critical-sized UHMWPE wear particles [12]. However, the molecular mechanisms of critical-sized UHMNWEP-ALN wear particles’ ability to influence the response of cells have not been clear. Additionally, the novel alginate (Alg)-encapsulated beads contained cell and critical-sized wear particles that have been fabricated to study the biological response of cells for a long term [19]. Therefore, the Alg bead was adopted as the cell reactor to evaluate the response of macrophages and osteoblasts to critical-sized UHMWPE-ALN wear particles in this study. Particularly, the molecular mechanisms of ALN in critical-sized UHMWPE-ALN wear particles effect on macrophages and osteoblasts were investigated in vitro.

In this study, there were principally two kinds of factors to affect the response of macrophages after being co-cultured with UHMWPE-ALN wear particles. One was due to the concentration of UHMWPE-ALN wear particles and the other was released ALN from UHMWPE-ALN wear particles. Fang et al. found that the proliferation of macrophages significantly reduced after being co-cultured with UHMWPE wear particles when the number ratio between the UHMWPE wear particles to macrophages was 10:1, while there was no significant difference in the proliferation of macrophages when the number ratio was 1:1 [16]. In this study, there was 1 mg wear particles per well, the number of which could be calculated as about 1 × 10^7^ according to the average particle size. Hence, the number ratio of wear particles to cells was 10:1 or so. Additionally, previous studies showed that bisphosphonates, such as ALN, could inhibit the proliferation and induce the death of macrophages when the concentration was within a certain range (10^−9^–10^−4^ M) [23,24]. It was calculated that the cumulative release of ALN in the first 14 d was within the above effective concentration range (up to 1.2 × 10^−5^ M) according to the profile of ALN release in our previous study [12]. As a result, a certain amount of wear particles and the effective concentration of ALN would synergistically reduce the proliferation of macrophages after being co-cultured with UHMWPE-ALN wear particles compared with those in the Alg + MPs + UHMWPE and Alg + MPs groups.

Early apoptosis of macrophages occurred due to the physical stimuli such as the morphology and hardness of UHMWPE wear particles when macrophages directly contact the surface of wear particles, then subsequently developed into late apoptosis. Studies found that ALN could reduce the formation of isoprenoid ester and inhibit the isoprenoid of protein by inhibiting intracellular mevalonate pathway, through which the formation of guanosine triphosphate reduced, the function of osteoclasts was inhibited, the cellular morphology changed, and the fold margin was lost. The above phenomena also hindered the secretory of H^+^ of the proton pump and the formation of an actin ring, which further increased the proportion of early apoptosis [25]. Moreau et al. investigated the apoptosis of macrophages after being exposed to different concentrations of ALN [26]. They found that ALN could cause the apoptosis of macrophages when the concentration was between 5 × 10^−6^ and 10^−4^ M [23,26]. The aforementioned concentration of ALN would feasibly cause the apoptosis of macrophages. In addition, bisphosphonates are generally divided into two categories: nitrogen-bisphosphonates and non-nitrogen bisphosphonates [23]. The ALN used in this study belongs to nitrogen-bisphosphonates, which can act on the cholesterol pathway by inhibiting diphosphate synthase in the mevalonate pathway, and ultimately lead to apoptosis of macrophages. Therefore, ALN released from UHMWPE-ALN wear particles significantly elevate the apoptosis rate of macrophages compared with that in the Alg + MPs + UHMWPE group in this study. Correspondingly, it reduced the proliferation of macrophages. More visual images (Figure 3) also demonstrated that there were more dead cells in the Alg + MPs + UHMWPE-ALN compared to those in the Alg + MPs + UHMWPE group and the Alg + MPs groups. As a result, the reduced proliferation and elevated early apoptosis rate of macrophages might serve as the effective approaches for restraining the biological response of macrophages, which would facilitate the reduction of wear particle-induced osteolysis.

It was found that macrophages can be activated and secrete a large number of inflammatory cytokines, e.g., TNF-α, IL-6, and IL-1β, which are three common inflammatory cytokines after being activated by phagocytosis of critical-sized UHMWPE wear particles [27,28]. They not only can recruit other inflammatory and immune cells, but also result in the recruitment, differentiation, and maturation of osteoclast precursors, and eventually lead to bone resorption and loosening of the implant [13]. This is a so-called cytokine pathway. Goodman et al. also found that the levels of cytokines secreted from macrophages were significantly increased after being co-cultured with UHMWPE wear particles [27]. In this study, the levels of cytokines, TNF-α and IL-6, secreted from macrophages was obviously increased after being co-cultured with critical-sized wear particles compared with those in Alg + MPs. Nevertheless, the lower levels of cytokines in Alg + MPs + UHMWPE-ALN were found compared those in Alg + MPs + UHMWPE after being co-cultured with critical-sized wear particles, which was due to the fact that ALN could reduce the production of inflammatory cytokines via the inhibition of phospho-nuclear factor -ĸB signaling pathway [25]. Similarly, the relatively lower gene expressions of TNF-α, IL-6, and IL-1β in Alg + MPs + UHMWPE-ALN was compared with those in Alg + MPs + UHMWPE although they were all significantly higher than those in the Alg + MPs group at the same time. It was deduced that the released ALN from critical-sized UHMWPE-ALN wear particles could inhibit the secretion of cytokines and the expression of a macrophage-related gene from macrophages and subsequently hamper the cytokine pathway, which is one of the main pathways of wear particle-induced osteolysis.

Secondly, the other pathway of wear particle-induced osteolysis is the RANKL/RANK signal pathway [29,30]. Osteolysis is generally considered a consequence of a disturbance in the mechanisms that govern the bone remodeling, mainly the communication between osteoclasts and osteoblasts [31]. RANKL is a transmembrane protein expressed by osteoblasts, stromal cells, and immune cells within the bone marrow. RANK is a receptor of trans-membrane protein and mainly expressed on the surface of macrophages, osteoclast precursors, and mature osteoclasts [13]. RANKL expressed on the membrane surface of osteoblasts could bind to RANK expressed on the membrane surface of the macrophage. After that, the intracellular RANKL/RANK signal pathway was initiated, the differentiation of osteoclasts was promoted, and the viability of mature osteoclasts was further enhanced, which eventually led to osteolysis [29,30]. It was found that the expression and combination of RANKL and RANK might significantly increase when the cells were exposed to UHMWPE wear particles according to the aforementioned pathway [30]. Hence, RANKL and RANK are essential for the differentiation of osteoclasts in the event of particle-induced osteolysis [32]. In this study, the relative gene expressions of RANKL and RANK in osteoblasts and macrophages in different wear particle groups were both significantly higher than that in the control group, which indicated wear particles up-regulated the gene expressions of RANKL and RANL in osteoblasts and macrophages, and subsequently enhanced the activity of the RANKL/RANK signal pathway. However, down-regulated gene expressions of RANKL and RANK in Alg + OBs + UHMWPE-ALN and Alg + MPs + UHMWPE-ALN would synergistically inhibit the differentiation and activation of osteoclasts, thereby reducing osteolysis. In addition, osteoprotegerin (OPG), a kind of soluble decoy receptor, could inhibit the differentiation of osteoclasts through its combination with RANKL instead of RANK. Therefore, OPG and the receptor activator of NF-ĸB ligand (RANKL) are the key molecules in the communications between osteoblasts and osteoclasts [29]. In addition, the gene expression of OPG of osteoblasts in Alg + OBs + UHMWPE-ALN was significantly higher than that in the Alg + OBs + UHMWPE group, although they both presented the down-regulated trend compared to that of Alg + OBs. As a result, OPG could combine with RANKL, hampering the RANKL/RANK signal pathway, interdict the differentiation of osteoclasts, and ultimately decrease the viability of mature osteoclasts [29]. In summary, the released ALN from UHMWPE-ALN wear particles could not only down-regulate the gene expression of RANKL and RANK but also up-regulate the gene expression of OPG to combine with RANKL, which could hamper the RANKL/RANK signal pathway and inhibit wear particle-induced osteolysis.

Furthermore, ALP activity is a significant feature of osteoblast differentiation, which is positively proportional with the differentiation of osteoblasts [33]. In this study, the up-regulated gene expression of ALP and OPG occurred in Alg + OBs + UHMWPE-ALN compared to that of Alg + OBs + UHMWPE. Particularly, it was found the up-regulated gene expression of ALN in the Alg + OBs + UHMWPE-ALN group after being co-cultured for 14 days. The up-regulated gene expression of ALP and OPG would facilitate osteogenesis, which was due to the released ALN in UHMWPE-ALN wear particles.

There is strong evidence that macrophages play the primary role in wear particle-induced osteolysis, which may secrete an array of cytokines after phagocytosing wear particles and further result in the maturation of osteoclasts through cytokine pathway [4]. Furthermore, UHMWPE wear particles could inhibit the osteogenic activity of osteoblasts and enhance the formation of osteoclasts through the receptor activator of nuclear factor kappa-B (RANK)/RANK ligand (RANKL) signal pathway, as a result, to induce osteolysis [13]. However, the present results showed that the released ALN or critical-sized UHMWPE-ALN wear particles phagocytosed by macrophages would obviously affect the biological response to macrophages and osteoblasts although UHMWPE-ALN wear particles were inevitably produced with the normal movement of the hip after UHMWPE-ALN was used as one of components of joint prosthesis replacement.

**Figure 8:**
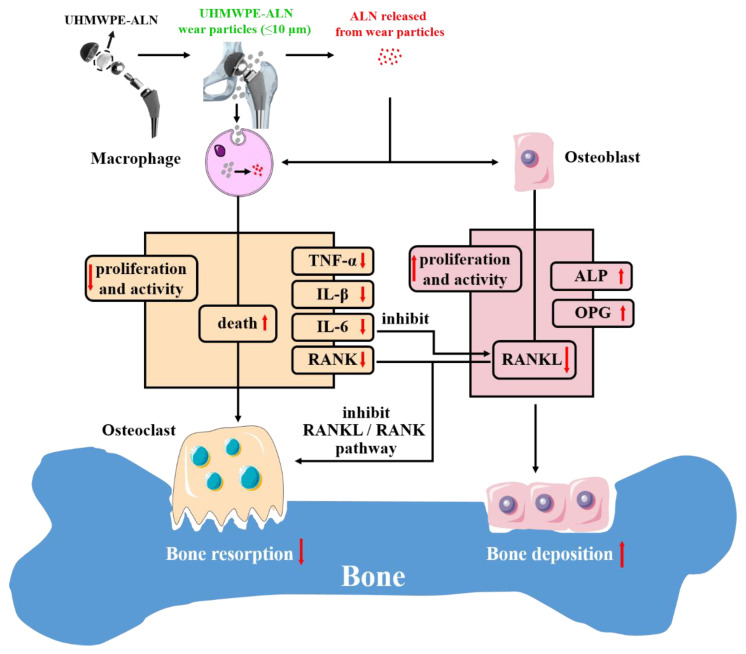
Mechanism of ALN in critical-sized UHMWPE-ALN wear particles to cells and osteolysis. Red upward arrow: increase, red downward arrow: decrease.

A primary mechanism of ALN in critical-sized UHMWPE-ALN wear particles to macrophages and osteoblasts was proposed as shown in Figure 8. For macrophages, UHMWPE-ALN wear particles would inhibit the proliferation and activity, promote the early apoptosis, and decrease the expression of a series of cytokines from macrophages. Concurrently, UHMWPE-ALN wear particles would directly promote the proliferation and ALP activity of osteoblasts [19], up-regulate the relative gene expression of ALP and OPG, and down-regulate that of RANKL. In addition, UHMWPE-ALN wear particles could inhibit the secretion of IL-6 macrophages, which subsequently inhibit the expression of RANKL on osteoblasts. The up-regulated gene expression of OPG would combine with RANKL instead of RANK, hamper the combination between RANKL and RANK, and ultimately interdict the differentiation and activation of osteoclasts. As a result, ALN in UHMWPE-ALN wear particles could alleviate the particle-induced osteolysis through the aforementioned interrelated synergies (mainly interfering with the cytokine and RANKL/RANK signal pathway), and potentially reduce the failure of the prosthesis and prolong its service life.

## 4. Materials and Methods

### 4.1. Preparation of Alg-Encapsulated Bead with Cells and Critical-Sized UHMWPE-ALN Wear Particles

Murine macrophage cell line RAW 264.7 was kindly provided by the Lab of Transplant Engineering and Immunology, West China Hospital, Sichuan University. Rat calvarial osteoblasts were isolated from a 10–15-day-old Sprague Dawley rat through a direct explants culture method [34]. Macrophages and osteoblasts were cultured in a high glucose Dulbecco’s Modified Eagle Medium (DMEM, Gibco, New York, NY, USA) and α-modified Eagle’s medium (α-MEM, Gibco, USA) supplemented with 10 vol.% fetal bovine serum (FBS, Gibco, USA), 100 unit/mL penicillin (Sigma, Burbank, CA, USA), and 100 unit/mL streptomycin (Sigma, USA) in an incubator at 37 °C with an atmosphere of 95% air-5% CO_2_.

According to our previous study, vacuum gradient filtration was used to obtain the critical-sized UHMWPE-ALN and UHMWPE wear particles, the mean sizes of which were 6.98 and 5.65 μm, respectively [12]. The above wear particles were sterilized by gamma irradiation at a dose of 25 kGy in a vacuum. A normal saline solution of 1.0 wt.% Sodium Alginate (Alg, Cat. No. 180947, Sigma-Aldrich, St. Louis, MO, USA) was sterilized by filtration. An Alg-encapsulated bead with cells and critical-sized UHMWPE-ALN wear particles, the cell reactor, was produced in sterile conditions using simple extrusion method described in our previous study [19]. Briefly, 40 mg of sterilized critical-sized UHMWPE-ALN wear particles and cells, with the number of macrophages and osteoblasts both being 2 × 10^7^ was suspended evenly in 20 mL of 1.0 wt.% Alg solution. It was then transferred into a 20-mL syringe connected to a syringe-driven pump and extruded into a 0.1 M calcium chloride (CaCl_2_) solution. The resulting cell reactors were allowed to harden in CaCl_2_ solution for 10 min and then washed thrice in 0.9 wt.% NaCl solution, which were named as Alg + MPs + UHMWPE-ALN and Alg + OBs + UHMWPE-ALN. Cell reactors contained macrophages or osteoblasts and critical-sized UHMWPE wear particles, and macrophages or osteoblasts alone were prepared according to the aforementioned description, which were named as Alg + MPs + UHMWPE, Alg + OBs + UHMWPE, and Alg + MPs and Alg + OBs.

Separately, 1 mL of the above cell reactor was cultured in each well of a 6-well cell culture plate supplemented with 5 mL of cell culture medium and incubated at 37 °C in an atmosphere of 95% air-5% CO_2_. Thus, 2 mg wear particles and 1 × 10^6^ cells or cells alone were contained per well as the control. The culture medium was replaced every 2 d in the experimental process.

### 4.2. Biological Evaluation of Critical-Sized UHMWPE-ALN Wear Particles on Macrophages Encapsulated in Cell Reactors

#### 4.2.1. Alamar Blue Assay

Alamar Blue assay (Invitrogen, Waltham, MA, USA) was used to determine the proliferation of macrophages after being co-cultured with critical-sized UHMWPE-ALN wear particles in cell reactors for 1, 4, 7, and 14 d [19]. Macrophages in cell reactors were incubated in 4 mL of DMEM with 10 vol.% Alamar Blue reagent for 4 h at 37 °C and 5% CO_2_ after a predetermined time. Then, 200 μL of cultured medium was transferred to a 96-well plate, the absorbance of which was measured at 570 and 600 nm with a microplate reader (µQUANT BIO-TEK Inc., Bio-Tek World Headquarters, Santa Clara, CA, USA). The data were expressed as relative proliferation rate according to following equations:

Reduction of Alamar Blue (%) = [(E600 × OD570) − (E570 × OD600)]/[(E570′ × OD600′) − (E600′ × OD570′)] × 100%(1)

Relative proliferation rate (%) = Reduction of Alamar Blue of different groups/Reduction of Alamar Blue in Alg + MPs beads on 1 d × 100%(2)

where E570 and E600 are the molar extinction coefficient of oxidized Alamar Blue at 570 nm (80,586) and 600 nm (117,216), and E570′ and E600′ are the molar extinction coefficient of reduced Alamar Blue at 570 nm (155,677) and 600 nm (14,652), respectively. OD570 and OD600 are optical densities of test wells at 570 and 600 nm, OD570′ and OD600′ are optical densities of the negative control at 570 and 600 nm, which only contained medium plus Alamar Blue but without cells.

#### 4.2.2. Cell Apoptosis Assay

The apoptosis of macrophages was determined by flow cytometry (FCM, Becton Dickinson, Franklin Lakes, NJ, USA) after being co-cultured with critical-sized UHMWPE-ALN wear particles in cell reactors for 2 and 7 d [35]. Briefly, cell culture medium was removed at each interval time, and 0.1 M citrate sodium solution was added to each well to dissolve cell reactors for 10 min. Macrophages were collected in tubes, centrifuged, and then washed twice with cold phosphate buffer saline (PBS). The cells were then stained with Annexin V-fluorescein isothiocyanate (FITC) and propidium iodide (PI) according to the protocol of Annexin V-FITC/PI apoptosis detection kit (Beyotime, Nantong, China) before being subjected to flow cytometry. Data was analyzed by FlowJo 7.6.1 software (Tree Star Inc., Ashland, OR, USA).

#### 4.2.3. Live-Dead Assay

The distribution of live and dead macrophages in different cell reactors was observed by confocal laser scanning microscope (CLSM system A1, Nikon Corp., Tokyo, Japan) after being incubated for 2 d [36]. After cell reactors were washed by PBS, 2 μM calcein acetoxymethyl ester (calcein-AM) and 4 μM PI dyes (Sigma, USA) were used to stain the live and dead cells at 37 °C for 20 min protected from light. Afterwards, cell reactors were washed by PBS and immediately scanned using CLSM.

#### 4.2.4. Cytokine Assay

Levels of tumor necrosis factor-alpha (TNF-α) and interleukin-6 (IL-6) in cell culture supernatant were measured using an enzyme-linked immunosorbent assay (ELISA) kit (BioSource, USA) after co-cultured macrophages and critical-sized UHMWPE-ALN wear particles were in cell reactors for 1, 7, and 14 d. Additionally, the addition of 1 μg/mL lipopolysaccharide (LPS, Sigma-Aldrich, USA) solution into the well that contained Alg + MPs beads served as the positive control [37]. The absorbance was read at 450 nm with a microplate reader. The concentrations of TNF-α or IL-6 were determined by a standard curve. The levels of TNF-α or IL-6 in per mL culture medium were normalized to the cell number of each group.

#### 4.2.5. Real-Time Polymerase Chain Reaction (RT-PCR) Analysis

Cell reactors were dissolved by 0.1 M citrate sodium solution for 10 min after being incubated for 1, 7, and 14 d, and subsequently macrophages were collected in tubes and washed by PBS. Total RNA was isolated using TRIzol reagent (Thermo, Waltham, MA, USA). The quantity and quality of freshly isolated RNA were determined by a spectrophotometer (Thermo, USA) with the absorbance at 260 nm/280 nm. Afterwards, cDNA was synthesized from 1 μg of RNA using reverse transcription reagent (Thermo, USA). RT-PCR was performed on CFX96 RT-PCR system (Bio-Rad Laboratories, Hercules, CA, USA) with iQ SYBR Green Supermix (Bio-Rad, USA). All procedures were conducted according to the manufacturer’s instructions. The results were normalized against the housekeeping gene glyceraldehyde-3-phosphate dehydrogenase (GAPDH) in the same sample. Primers were designed using Primer Express based on the sequences from the Genbank database and were synthesized by the Sangon Biotech (Shanghai) Co., Ltd., Shanghai, China. The primer sequences of selected genes of macrophages for RT-PCR are shown in Table 1. The relative expression levels of genes were calculated using the method by normalizing with glyceraldehyde-3-phosphate dehydrogenase (GAPDH) housekeeping gene expression [38].

### 4.3. Biological Evaluation of Critical-Sized UHMWPE-ALN Wear Particles on Osteoblasts Encapsulated in Cell Reactors

#### 4.3.1. Alkaline Phosphatase (ALP) Activity Assay

The differential function of encapsulated osteoblasts was assessed by monitoring the ALP activity (ALP detection kit, Beyotime, China) after being co-cultured with critical-sized UHMWPE-ALN wear particles for 4, 7, and 14 d [39]. Osteoblasts in cell reactors of different groups were dissolved by 0.1 M citrate sodium for 10 min prior to being collected. ALP activity was detected according to the manufacturer’s instruction and normalized to the total protein content (U/gprot) measured by the bicinchoninic acid (BCA) protein assay kit (Beyotime, China).

#### 4.3.2. RT-PCR Analysis

The relative gene expression levels of osteoblasts were measured by RT-PCR after cells were co-cultured with critical-sized UHMWPE-ALN wear particles in cell reactors for 4, 7, and 14 d. The primer sequences of selected genes are listed in Table 2, including ALP, RANKL, OPG, and GAPDH. The protocol was similar with that in Section 4.2.5.

### 4.4. Statistical Analysis

Each experiment was performed thrice. All quantitative data are presented as the mean ± standard deviation (SD). Statistical significance was determined by one-way analysis of variance (ANOVA) followed by Tukey’s test. A value of *p* < 0.05 was considered as statistically significant.

## 5. Conclusions

In this study, the molecular impact of critical-sized UHMWPE-ALN wear particles on both of macrophages and osteoblasts were investigated by Alg-encapsulated cell reactor. Compared with the critical-sized UHMWPE wear particles, critical-sized UHMWPE-ALN wear particles inhibited the proliferation and activity of macrophages, promoted the early apoptosis, and suppressed the secretion of TNF-α and IL-6. Moreover, critical-sized UHMWPE-ALN wear particles down-regulated macrophage-relative gene expressions of TNF-α, IL-6, and IL-1β and RANK. Critical-sized UHMWPE-ALN wear particles promoted the ALP activity of osteoblasts, and down-regulated the gene expression of RANKL compared with those of UHMWPE wear particles. Furthermore, they up-regulated the osteoblast-relative gene expression of OPG, which could hamper the combination between RANKL and RANK, and ultimately interdict the differentiation and activation of osteoclasts. There were mainly two approaches of critical-sized UHMWPE-ALN wear particles to effect on the biological response of macrophages and osteoblasts, one of which was cytology and the other was cytokine signal pathway. The former would mainly inhibit the proliferation of macrophages and elevate the ALP activity of osteoblasts. The latter would potentially interdict the differentiation and activation of osteoclasts by the cytokine and RANKL/RANK signal pathway.

## Figures and Tables

**Table 1:** The primer sequences of macrophages for RT-PCR.

Gene	Direction	Sequence(5′-3′)
TNF-α	Forward	AAGTTCCCAAATGGCCTCCC
	Reverse	TGGTTTGCTACGACGTGGG
IL-6	Forward	TAGTCCTTCCTACCCCAATTTCC
	Reverse	TTGGTCCTTAGCCACTCCTTC
IL-1β	Forward	GCCACCTTTTGACAGTGATGA
	Reverse	ATGTGCTGCTGCGAGATTTG
RANK	Forward	CCAGGAGAGGCATTATGAGCA
	Reverse	ACTGTCGGAGGTAGGAGTGC
GAPDH	Forward	GGACCAGGTTGTCTCCTGTG
	Reverse	CATTGAGAGCAATGCCAGCC

**Table 2:** The primer sequences of osteoblasts for RT-PCR.

Gene	Direction	Sequence(5′-3′)
ALP	Forward	GGAACGGATCTCGGGGTACA
	Reverse	ATGAGTTGGTAAGGCAGGGT
RANKL	Forward	GGGGGAGCACTAAGAACTGG
	Reverse	GTTGGACACCTGGACGCTAA
OPG	Forward	CAGTGTGCAACGGCATATCG
	Reverse	CCAGGCAAGCTCTCCATCAA
GAPDH	Forward	GGACCAGGTTGTCTCCTGTG
	Reverse	CATTGAGAGCAATGCCAGCC

## Data Availability

Not applicable.
